# Body weight, eating practices and nutritional knowledge amongst university nursing students, Eastern Cape, South Africa

**DOI:** 10.4102/phcfm.v4i1.323

**Published:** 2012-08-21

**Authors:** Violet L. van den Berg, Alice P. Okeyo, Andre Dannhauser, Mariette Nel

**Affiliations:** 1 Department of Nutrition and Dietetics, University of the Free State, South Africa; 2 Department of Biostatistics, University of the Free State, South Africa

## Abstract

**Background:**

Health care workers need to be equipped to deal with the increasing obesity and obesity-related morbidity occurring in developing countries.

**Objectives:**

To assess weight status, eating practices and nutritional knowledge amongst nursing students at the University of Fort Hare, Eastern Cape.

**Method:**

A cross-sectional descriptive survey was conducted on 161 undergraduate (51 male and 110 female) students of the Department of Nursing Sciences at the University of Fort Hare. Body mass index, waist and hip circumferences and waist hip ratio were determined. Nutritional knowledge and eating practices were investigated by structured interviewer-administered questionnaires.

**Results:**

Statically, 49.7% were overweight or obese (58.2% of the females; 31.4% of the males) and 65.2% had waist circumferences putting them at risk for non-communicable diseases. Most did not meet the recommendations for intakes from the vegetable group (97.5% ate <3 servings per day), the fruit group (42.2% ate <2 servings per day), and the dairy group (92.6% ate <2 servings per day); whilst 78.3% ate ≥4 serving per day of sugar or sweets. Most consumed margarine, oil or fat (68.3%), sugar (59.0%) and bread (55.9%) daily, but few reported daily intakes of vegetables (12.4%), fruit (23.6%), fruit juice (21.2%) and milk (15.6%). Fewer than 50% knew the recommended intakes for vegetables, fruit, dairy, starchy foods and meat or meat alternatives.

**Conclusions:**

These nursing students had a high prevalence of overweight and obesity, poor eating habits and inadequate knowledge on key nutrition issues, which may impact negatively on their efficacy as future health ambassadors to the public.

## Abstrait

**Poids corporel, pratiques alimentaires et connaissances en matière de nutrition parmi les étudiants en sciences infirmières, Cap Oriental, Afrique du Sud**

**Contexte:**

Les travailleurs de la santé doivent être équipés pour faire face à l'augmentation de l'obésité et de la morbidité liée à l'obésité dans les pays en développement.

**Objectifs:**

Évaluer le statut pondéral, les pratiques alimentaires et les connaissances en matière de nutrition des étudiants en sciences infirmières à l'Université de Fort Hare, Cap oriental.

**Méthode:**

Une enquête transversale descriptive a été réalisée auprès de 161 étudiants de premier cycle (51 hommes et 110 femmes) du département des sciences infirmières à l'Université de Fort Hare. L'indice de masse corporelle, le tour de taille et de hanches et le rapport taille-hanche ont été déterminés. Les connaissances en matière de nutrition et les pratiques alimentaires ont été examinées à partir de questionnaires administrés par un enquêteur.

**Résultats:**

Statistiquement, 49.7% étaient en surpoids ou obèses (58.2% des femmes; 31.4% des hommes) et 65.2% avaient un tour de taille augmentant le risque de souffrir de maladies non contagieuses. La plupart ne respectent pas les recommandations en termes d'apports alimentaires provenant du groupe alimentaire des légumes (97.5% mangeaient moins de 3 portions par jour), du groupe alimentaire des fruits (42.2% mangeaient moins de 2 portions par jour), et du groupe alimentaire des produits laitiers (92.6% mangeaient moins de 2 portions par jour); alors que 78.3% mangeaient 4 portions par jour de sucre ou de bonbons ou plus. La plupart consommaient de la margarine, de l'huile ou de la graisse (68.3%), du sucre (59.0%) et du pain (55.9%) tous les jours, mais moins de 50% mentionnaient un apport en légumes, fruits, produits laitiers, féculents et viande ou substituts de viande.

**Conclusions:**

Ces étudiants en sciences infirmières présentaient un taux de prévalence de surpoids et d'obésité élevé, de mauvaises habitudes alimentaires et une méconnaissance des points nutritionnels clés.

## Introduction

### Key focus

South Africa,^1^ like other sub-Saharan African countries,^2^ has a double burden of disease. Undernutrition, with its outcomes of growth failure, stunting and micronutrient deficiencies, still occurs amongst children in developing communities; whilst overnutrition, leading to overweight and obesity, and non-communicable diseases (NCD), including hypertension, cardiovascular diseases and type 2 diabetes, is reaching epidemic proportions amongst adults in the same communities.^1, 2^

### Background and Trends

The South African Demographic and Health Survey (SADHS) 2003 found that 29.8% of South African adult men and 54.7% of adult women (the four main races in the country combined) were either overweight or obese (had a body mass index [BMI] of 25–30kg/m^2^ or >30kg/m^2^, respectively).^3^ The mean waist circumference for South African women in the SADHS was found to be 82.7cm^3^, which is above the cut-off point for sub-Saharan females (>80cm)^4^ that constitutes an increased risk for insulin resistance and the metabolic syndrome. A relatively high percentage of urban African women (39%)^3^ also had a waist circumference of >88cm, classified as a substantial risk for NCD.^5^ The health impact of these findings is emphasised by the South African National Burden of Disease Study which estimated that NCD, associated with a high BMI, accounted for 37% of all deaths in the country in 2000.^6^

The etiology of obesity involves a complex interplay between genetics and environmental or lifestyle factors.^7, 8^ Eating practices associated with the global obesity epidemic include increased consumption of energy-dense, but nutrient-poor foods;^7, 9^ low consumption of dairy products^10^, fruits and vegetables^9^, skipping breakfast^11^; and insufficient physical activity.^7, 8^ South Africa, like other developing countries, has been undergoing a transition from traditional high fibre, low fat diets, to typical Western diets that are high in fat, sodium and added sugars, and low in unrefined carbohydrate, dairy, fruits and vegetables; and from traditionally more active lifestyles, to more sedentary practices.^1, 12, 13^ These trends have been associated with the high prevalence of obesity amongst Black South African people, particularly women living in urban areas.^3, 12, 13^

Studies have shown that counselling interventions are effective in reducing risk and burden of disease in adults with hyperlipidemia and other risk factors for cardiovascular diseases.^14^ Since the public views primary care providers as valuable sources of nutritional guidance and lifestyle advice to prevent and treat NCD, health care professionals play a key role in this regard through patient education.^15, 16, 17^ However, studies identify significant barriers which prevent health care workers from offering dietary support; these include lack of time, of teaching materials, of nutritional knowledge and of confidence, on the part of the provider.^18, 19^ Moreover, it seems from a recent systematic review, that health care professionals are more likely to discuss weight, diet and lifestyle issues with their patients and use strategies to prevent obesity in patients, if they themselves have a normal BMI.^20^ No published studies to date have addressed these issues amongst South African health care professionals.

### Objectives

The training of health care professionals at colleges and universities offers the opportunity to develop sound knowledge, attitudes and practices regarding nutrition and weight. This study was undertaken to assess the body weight status, eating practices and nutritional knowledge of nursing students at the University of Fort Hare, and to investigate possible associations between these parameters.

### Research significance

Nurses play a key role in addressing the emerging epidemic of overweight and obesity in South Africa on a primary level through patient education. Studies show however, that health care professionals are more likely to council patients on weight, diet and lifestyle issues if they themselves have a normal body mass index. No published studies to date have addressed these issues amongst South African health care professionals. This study was undertaken to assess the weight status, eating practices and nutritional knowledge of nursing students at the University of Fort Hare, Eastern Cape.

The baseline information gathered in this study may highlight knowledge, attitude or practice gaps which need to be addressed in order to empower nurses towards efficient diet and lifestyle counselling in South Africa.

## Ethical considerations

All procedures of this study adhered to the Declaration of Helsinki on ‘Ethical Principles for Medical Research Involving Human Subjects.’ The study protocol was approved by the Ethical Committee of the Faculty of Health Sciences, University of the Free State (ETOVS NR 37/07). Permission to carry out the research was obtained from the Head of the Department of Nursing Sciences, University of Fort Hare. The study was conducted in 2007.

### Potential benefits and hazards

Participation in the study involved no risk to the participants. In cases where health risks were identified in a subject during the study, participants received free dietetic advice and counselling. All information in the study was kept strictly confidential. During the reporting of the results the focus was strictly on group trends so that individual information remained confidential and anonymous within the study.

### Recruitment procedures

All 200 nursing students enrolled at the institution, were contacted and informed of the study. No student was under any obligation to participate in the study, and those contacted were informed (orally and in writing) that their participation would be voluntary, and that they would not be penalized or lose benefits if they refused to participate, or wanted to withdraw from the study at any time. They were also informed that participants would not carry any costs and no participant would receive any compensation.

### Informed consent

All participants were pre-informed in writing about the purpose of the study and of the procedures to be used during the research. Students, who agreed to these terms signed letters of consent. Participants retained the written information sheets.

## Methods

### Population and setting

Two hundred full-time undergraduate students of 18 years and older, from the Department of Nursing Sciences, University of Fort Hare, Eastern Cape, South Africa were contacted – their names were obtained from the admissions list of the institution. A total of 161 students (51 male and 110 female) gave consent and were included in the study.

### Design

A cross-sectional survey was conducted.

### Procedure

The diagnosis of overweight or obesity and the risk for NCDs was based on the Body Mass Idex (BMI) and waist circumference, respectively, as recommended by both the International and Southern African Associations for the Study of Obesity (IASSO and SASSO).^5^ Waist-hip-ratios were also included as a measure of fat distribution.^21^ All measurements were taken in light clothing and without shoes by the same trained researcher, using standardised techniques.^4, 21^ The average of three measurements was recorded.

Structured interviews with the participants were performed by the main researcher, who had been trained in nutrition at the University of the Free State. Interviews were conducted in a room at the Department of Nursing Sciences of the University at Fort Hare, and food photos and packaging were used to assist participants in recalling portion sizes and food choices. Eating practices were established by 24-hour recalls obtained during structured interviews with the participants. As a single 24-hour recall does not adequately represent usual food intake, three recalls (for Tuesdays, Thursdays and Saturdays) were recorded for each participant, and the average daily intakes were calculated. The United States Agricultural Department's Food Guide Pyramid 1992 (USAD-FGP)^22^ was used to evaluate the food intake, as this tool is familiar to South African students in health care, and quantifies the recommended daily number of portions to be consumed from each food group (as opposed to the South African Food-Based Dietary Guidelines [SAFBDG]^23^). The Dietary Guidelines for Americans 2010^24^ was also used to interpret fat and sugar intakes (not included in the USDA-FGP 1992). Average daily energy intakes were quantified from the data collected in the 24-hour recalls, using a standard exchange list based on the recommendations of the American Dietetic Association^25^, with portion sizes quantified from the South African Medical Research Council's (MRC) Food Composition Tables^26^ and the SA MRC Food Quantity Tables.^27^ During the same interview, a food frequency questionnaire, which was adapted to reflect food choices of students and used to determine how often a particular food was consumed, was also administered.

Nutritional knowledge was recorded during structured interviews with the participants in their language of choice, using a questionnaire based on the recommendations of the USDA-FGP^22^, the Dietary Guidelines for Americans 2010^24^ and the SAFBDG.^23^ Questions were asked regarding the food group to be eaten most frequently; the food group to be eaten the least; recommended number of daily servings from each food group; foods with high fat, sugar and fibre content; foods with low fat, sugar and fibre content; and foods high in vitamin C and beta carotene. Students who had an overall score of more than 50% on the knowledge questionnaire were regarded as knowledgeable, whilst individuals who scored less than 50% were regarded as less knowledgeable. Students were also asked to identify their sources of nutritional information – be it their parents, their friends, the media or a school or institution of learning.

### Analysing

A statistical data analysis was performed by the Department of Biostatistics of the Faculty of Health Science of the University of the Free State, and was generated by SAS^®^ software (copyright, SAS Institute – SAS and all other SAS Institute Inc. products or service names are registered trademarks or trademarks of SAS Institute Inc., NC, USA.) Categorical data was presented as frequencies and percentages, and continuous data as medians and percentiles. To compare the underweight, normal weight and overweight or obese groups, 95% confidence interval [CI] for median differences or percentage differences were used.

## Results

### Socio-demographic status of the nursing students

The final sample of 161 students had a median age of 24.9 years (ranging from 18 to 42 years) and consisted of two-thirds females (Table 1). There were 27.3%, 26.1%, 19.9%, and 26.7% first, second, third and fourth year students, respectively. The majority (96.3%) of the students were Black students; most of whom (87.6%) were single; most (67.7%) resided at the university hostels; and for most (42.2%) students their permanent place of residence was in rural areas, followed by township places of residence (37.3%). Those residing at the university hostels were responsible for procuring or preparing their own meals, as the campus does not offer a food service.

**TABLE 1:** Socio-demographic status of nursing students (*N* = 161).

Group	%	%
**Gender**		
Males	51	31.7
Females	110	68.3
**Ages**		
Lower Quartile (25th)	22.3	-
Median	24.9	-
Upper Quartile (75th)	28	-
**Ethnicity**		
Black	155	96.3
White	2	1.2
Coloured	4	2.5
**Academic level of study**		
First year	44	27.3
Second year	42	26.1
Third year	32	19.9
Fourth year	43	26.7
**Marital Status**		
Married	20	12.4
Single	141	87.6
**Current place of Residence**		
University hostels	109	67.7
Private rental	28	17.4
At home	23	14.3
Other	1	0.6
**Permanent place of residence**		
Urban Town	33	20.5
Township	60	37.3
Rural areas	68	42.2

*n*, Given as number of nursing students.

### Body weight status of the nursing students

Based on the BMI-classification for adults,^5, 21^ 46.7% of the participants were classified as either overweight (31.7%) or obese (15.0%) (Table 2). Overweight and obesity, respectively, were also more common amongst female students (36.4% and 21.8%) than males (21.6% and 9.8%). The waist circumference-values indicated that 65.2% of the participants were *at risk* for insulin resistance, the metabolic syndrome and thus for NCD, as defined by IASSO.^5^ Based on waist circumference and waist-hip-ratio^21^, 38.5% and 37.3% of all the participants respectively, were *at substantial risk* for NCD as defined by IASSO. (Table 2)

**TABLE 2:** Body weight status and fat distribution of nursing students (*N* = 161).

Categories (BMI kg/m^2^)†	*n*	%
**Male and female combined (*n* = 161)**		
Underweight (BMI < 18.5 kg/m^2^)	7	4.4
Normal weight (BMI 18.5–24.9 kg/m^2^)	74	45.9
Overweight (25–29.9 kg/m^2^)	51	31.7
Obese (BMI ≥ 30 kg/m^2^)	29	15.0
**Male (*n* = 51)**		
Underweight (BMI < 18.5 kg/m^2^)	5	9.8
Normal weight (BMI 18.5–24.9 kg/m^2^)	30	58.8
Overweight (25–29.9 kg/m^2^)	11	21.6
Obese (BMI ≥ 30 kg/m^2^)	5	9.8
**Female (*n* = 110)**		
Underweight (BMI < 18.5 kg/m^2^)	2	1.8
Normal weight (BMI 18.5–24.9 kg/m^2^)	44	40.0
Overweight (25–29.9 kg/m^2^)	40	36.4
Obese (BMI ≥ 30 kg/m^2^)	24	21.8
**Waist Circumference (WC) Categories (*n* = 161)**		
Ideal category (< 94 cm male & < 80 cm female)	56	34.8
Increased risk for chronic diseases of lifestyle category‡ (94.0 - 101.9 cm male & 80.0 - 87.9 cm female)	43	26.7
Substantially increased chronic diseases of lifestyle category‡ ≥ 02 cm male & ≥ 88 cm female	62	38.5
At risk for insulin resistance and the metabolic syndrome§ ≥ 94 cm male & ≥ 80 cm female	105	65.2
Waist Hip Ratio (WHR) Categories (*n* = 161)		
Ideal category < 0.9 males & < 0.8 female	101	62.7
Increased risk for chronic diseases of lifestyle category†† ≥ 0.9 for male & ≥ 0.8 for female	60	37.3

† WHO BMI classification - Southern African Society for the Study of Obesity. Guidelines for the Prevention and Management of Overweight and Obesity in South Africa [homepage on the internet]. 2003 [cited 2011 Apr. 28]. Available from: http://www.hypertension.org.za/ClientFiles/Guidelines/SASSO%20Guidelines.pdf

‡ As defined by: Southern African Society for the Study of Obesity. Guidelines for the Prevention and Management of Overweight and Obesity in South Africa [homepage on the internet]. 2003 [cited 2011 Apr. 28]. Available from: http://www.hypertension.org.za/ClientFiles/Guidelines/SASSO%20Guidelines.pdf

§ As defined by the Alberti KG, Eckel RH, Grundy SM, et al. Harmonizing the metabolic syndrome. A joint interim statement of the International Diabetes Federation Task Force on Epidemiology and Prevention; National Heart, Lung, and Blood Institute; American Heart Association; World Heart Federation; International; Atherosclerosis Society; and International Association for the Study of Obesity. Circulation. 2009;120:1640—1645.

†† As defined by Bray GA. Pathophysiology of obesity. Am J Clin Nutr. 1992;55:488S–494.

*n*, Given as number of nursing students.

### Eating practices of nursing students

The majority of participants (59%) ate three meals a day, 23.6% reported eating fewer than three meals per day, and all of them (161) indicated that lunch was the meal that they most frequently skipped. The usual daily food intake obtained from the average of three 24-hour recalls and compared to the recommendations of the USDA-FGP^22^ and the Dietary Guidelines for Americans 2010.^24^ More than 9 out of 10 participants did not meet the minimum daily requirements for vegetables (97.5%) and dairy or dairy products (92.6%), and 42.2% did not consume the minimum recommended amount of fruit. Most participants (83.2%) consumed adequate amounts of bread, cereal, rice and pasta daily, whilst 13.6% consumed more than the recommended 11 servings from this food group per day. Most participants (80.8%) reported intakes above the minimum recommendations (two to three servings) for meat and meat substitutes. Most participants (78.3%) also consumed 4 or more teaspoons (40g+) of added sugar and/or sweets per day; whilst half (50.3%) the participants consumed more than 20g of added fats and oils per day.

Most participants reported that they consumed margarine, oil or fat (68.3%), sugar (59.0%) and bread (55.9%) daily, but few consumed vegetables (12.4%), fruit (23.6%), fruit juice (21.2%), and dairy or dairy products (15.6%) on a daily basis (Table 4). A large number (77.0%) of the participants did not consume low fat or skim milk. Most participants (73.9%) reported not using any alcohol and less than one per cent reported daily consumption of alcohol (Table 4). Self-reported alcohol intakes (Table 3) of most participants (98.8%) were within the prudent guidelines of two or fewer drinks per day.^28^

**TABLE 3:** Usual daily food intake of nursing students (*N* = 161).

Food groups	Recommendations†	*n*	%
**Breads, Cereal, Rice and Pasta**			
Below recommendations	< 6 servings per day	6	3.7
Within recommendations	6–11 servings per day	134	83.2
High intakes	>11 servings per day	21	13.1
**Vegetables**			
Below recommendations	< 3 servings per day	157	97.5
Within recommendations	3–5 servings per day	4	2.5
**Fruits**			
Below recommendations	< 2 servings per day	68	42.2
Within recommendations	2–4 servings per day	78	48.5
High intakes	>4 servings per day	15	9.3
**Milk and milk products**			
Below recommendations	< 2 servings per day	149	92.6
Within recommendations	2–3 servings per day	12	7.5
**Meat and meat alternatives**			
Below recommendations	< 2 servings per day	5	3.1
Within recommendations	2–3 servings per day	26	16.2
High intakes	>3 servings per day	130	80.8
**Fats and oils**			
-	≤ 4 servings (20g) ‡ per day	80	49.7
-	>4 servings (20g) per day	81	50.3
**Sweets and sugar**			
-	≤ 4 servings (40g) per day	35	21.7
-	> 4 servings (40g) per day	126	78.3
**Alcohol**			
Recommended allowance	≤ 2 units per day§	159	98.8
Above recommendation	>2 units per day	2	1.2

† Evaluated according to the recommendations of the American Department of Agriculture's Food Guide Pyramid 1992 (Shaw A, Fulton L, Davis C, Hogbin M. Using the Food Guide Pyramid: A Resource for nutrition educators. U.S. Department of Agriculture Food, Nutrition, and Consumer Services Center For Nutrition Policy and Promotion. Accessed: 10 May 2011. Available form: http://www.nal.usda.gov/fnic/Fpyr/guide.pdf).

‡ Based on the recommendation of the Dietary Guidelines for Americans 2010, that people with an average daily intake of 5000-6000kJ should consume 20g or less of added fats and oils (U.S. Department of Agriculture U.S. Department of Health and Human Services. Dietary Guidelines for Americans 2010:79, Available at: www.dietaryguidelines.gov).

§ National Institutes of Health, National Heart, Lung, and Blood Institute National High Blood Pressure Education Program: The Seventh Report of the Joint National Committee on Prevention, Detection, Evaluation, and Treatment of High Blood Pressure, NIH Publication No.04-5230, 2004.

*n*, Given as number of nursing students.

**TABLE 4:** Frequency of food consumption of nursing students (*N* = 161).

Food	Do not eat		Eat monthly		Eat daily	
		
	*n*	%	*n*	%	*n*	%
Sweets and/or chocolates	7	4.4	124	77.0	30	18.6
Chips (crisps)	17	10.6	134	83.2	10	6.2
Cakes and/or biscuits	19	11.8	138	85.7	4	2.5
Cooldrinks	13	8.1	116	72.1	32	19.8
Cremora	78	48.4	60	37.3	23	14.3
Coffee	43	26.7	84	52.2	34	21.1
Tea	63	39.1	70	43.5	28	17.4
Sugar	9	5.6	57	35.4	95	59.0
Full cream milk	31	19.3	110	68.3	20	12.4
Low fat/skim milk	124	77.0	32	19.9	5	3.1
Eggs	9	5.6	132	82.0	20	12.4
Peanut Butter	69	42.9	91	56.5	1	0.6
Soya minced/legumes (baked beans, dried beans, peas and lentils)	74	46.0	86	53.4	1	0.6
Chicken	6	3.7	144	89.5	11	6.8
Red meat	18	11.2	142	88.2	1	0.6
Fish	33	20.5	125	77.6	3	1.9
Bread	0	0.0	71	44.1	90	55.9
Cooked porridge	55	34.2	106	66.8	0	0.0
Cereal (Morevit/Pronutro)	42	26.1	113	70.2	6	3.7
Samp and/or mielie rice	32	19.9	123	76.4	6	3.7
Margarine and/or oil or fat	1	0.6	50	31.1	110	68.3
Fruit juice	16	9.9	111	68.9	34	21.2
Fruit	0	0.0	123	76.4	38	23.6
Vegetables	4	2.5	137	85.4	20	12.4
Salt and/or stork or Royco	0	0.0	22	13.7	139	86.3
Alcohol	119	73.9	41	25.5	1	0.6

*n*, Given as number of nursing students.

### Nutritional knowledge of nursing students

The majority of the participants (Table 5) did not know that starch should be eaten the most (68.3%), but did know that fat or oil and sugar or sweets should be eaten the least (75.2%). The majority of the participants also did not know the daily recommended number of servings that should be consumed from the bread, cereal, rice and pasta group (85.7%), the vegetables group (54.7%), the milk or yoghurt and cheese group (60.2%), or the meat, poultry, fish, dry beans, eggs and nuts group (57.1%). Almost half the participants did not know the daily recommended number of servings of fruit that should be consumed (44.7%), or that peanut butter is high in fat (49.7%); but most knew which foods have a high fibre content (92.6%); that fried chicken has a high fat content (97.5%), and that carrot is the best source of beta carotene (96.3%).

**TABLE 5:** Responses to nutritional knowledge questions (*N* = 161).

Nutritional knowledge tested†	Answered incorrectly		Answered correctly	
	
	*n*	%	*n*	%
Which food group should be eaten the most	110	68.3	51	31.7
Which food group should be eaten the least	40	24.8	121	75.2
Daily recommended number of servings for fruits	72	44.7	89	55.3
Daily recommended number of servings of milk and/or yoghurt and cheese	97	60.2	64	39.8
Daily recommended number of servings of bread, cereal, rice and pasta	138	85.7	23	14.3
Daily recommended number of servings of vegetables	88	54.7	73	45.3
Daily recommended number of servings of meat, poultry, fish, dry beans, eggs and nuts	92	57.1	69	42.9
Which foods are high in fibre	12	7.5	149	92.6
Fried chicken as food with high fat content	4	2.5	157	97.5
Peanut butter as food with high fat content	80	49.7	81	50.3
Foods which are best sources of β carotene	6	3.7	155	96.3

† Questions and answers based on:

• Shaw A, Fulton L, Davis C, Hogbin M. Using the Food Guide Pyramid: A Resource for nutrition educators. U.S. Department of Agriculture Food, Nutrition, and Consumer Services Center For Nutrition Policy and Promotion [home page on the internet]. No date.[cited: 2011 May 10. Available form: http://www.nal.usda.gov/fnic/Fpyr/guide.pdf

• The South African Food Based Dietary Guidelines [home page on the internet]. 2011cited 2011 May 10]. Available from: http://www.sahealthinfo.org/nutrition/foodbasedsept2001.pdf

• U.S. Department of Agriculture and U.S. Department of Health and Human Services. Dietary Guidelines for Americans, 2010. 7th Edition, Washington, DC: U.S. Government Printing Office, 2010:79.

*n*, Given as number of nursing students.

Almost two-thirds of the participants (65.4%) had an overall score of more than 50% on the nutritional knowledge questionnaire and were classified as knowledgeable, whilst 34.6% of the participants had an overall score of less than 50% and were classified as not knowledgeable.

Most participants listed the media (47.8%) and school or institution of teaching (35.4%) as their sources of nutritional information. Parents (13.7%) and friends (22.9%) did not rank high as sources of information.

### Association between eating practices and body weight

Median energy intakes were significantly higher for men (6 333 kJ) than for woman (5 543 kJ) (95% CI for median difference [236.7; 970.0]). No statistical differences were found between BMI or waist circumference categories with regard to gender, place of residence, number of meals per day, breakfast skipping, or median energy or macronutrient intakes. (Note however, that there were only seven participants in the underweight category).Significantly more underweight than overweight or obese participants, consumed fewer than six servings from the starch group daily [95% CI: 0.1%; 50.1%]. Significantly fewer underweight participants also consumed chips (crisps) on a daily basis than both normal weight [95% CI: -61.5%; -1.7%], and overweight or obese participants [95% CI: -66.0%; -6.8%]. Significantly fewer underweight participants also (57%) consumed sweets or chocolates on a daily basis than overweight or obese participants (90%) [95% CI: -65.3%; -4.5%] (Table 6).

**TABLE 6:** Association between Body Mass Index and frequency of food consumption (*N* = 161).

Foods	Underweight†		Normal weight‡		Overweight/obese§	
		
	%	*n*	%	*n*	%	*n*
**Sweets and chocolates**						
Never eats	0	0	0	0	0	-
Eats monthly	42.9	3	14.9	11	10	8
Eats daily	57.1	4	85.1	63	90	72
**Chips (crisps)**						
Never eats	0	0	0	0	1.3	1
Eats monthly	71.4	5	32.4	24	26.3	21
Eats daily	28.6	2	67.6	50	72.5	58
**Cakes and/or biscuits**						
Never eats	0	0	5.4	4	6.3	5
Eats monthly	14.3	1	32.4	24	40.0	32
Eats daily	85.7	6	62.2	46	53.8	43
**Cool drinks**						
Never eats	0	0	0	0	0	-
Eats monthly	42.9	3	40.5	30	47.5	38
Eats daily	57.1	4	59.5	44	52.5	42
**Full cream milk**						
Never eats	0	0	5.4	4	3.8	3
Eats monthly	85.7	6	82.4	61	71.3	57
Eats daily	14.3	1	12.2	9	25.0	20
**Low fat and/or skim milk**						
Never eats	14.3	1	37.8	28	42.5	34
Eats monthly	28.6	2	45.9	34	42.5	34
Eats daily	57.1	4	16.2	12	15.0	12

*n*, Given as number of nursing students.

† *n* = 7.

‡ *n* = 74.

§ *n* = 80.

Significantly more overweight or obese (25%), than normal weight (12.2%), participants consumed full cream milk on a daily basis [95% CI: -24.7%;-0.4%]. On the other hand, more underweight participants (57.1%) than both overweight or obese participants (15%) [95% CI: 8.7%; 69.9%], and normal weight participants (16.2%) [95% CI: 7.3%; 68.8%] consumed low fat or skim milk on a daily basis.

### Association between nutritional knowledge and body weight

Significantly more underweight participants (85.7%) than both normal weight (35.1%) [95% CI: 11.8%; 65.9%) and overweight or obese participants (40%) [95% CI: 7.1%; 61.1%] knew how many dairy servings should be eaten daily. More normal weight participants (20.3%) than overweight or obese participants (8.8%) [95% CI: 0.3%; 22.9%] knew the daily recommended servings for bread, cereal, rice and pasta; whilst more [95% CI: 0.7; 22.5%] normal weight (18.9%) than overweight (7.5%) participants knew that carrots are high in fibre. Significantly more underweight (28.6%) than overweight or obese participants (3.8%) [95% CI: 3.4%; 60.4%] thought that hamburgers are high in fibre. Significantly more underweight (14.3%) than overweight or obese participants (0%) [95% CI: 1.7%; 51.3%] knew that oranges are high in vitamin C. More overweight or obese (95%) participants [95% CI: -59.2%; -2.0%] than underweight (71.4%) participants knew that carrots are a good source of ß-carotene.

Participants with adequate nutritional knowledge (who scored ≥50% on the knowledge questionnaire) did not eat significantly different from those with inadequate nutritional knowledge (who scored <50) regarding any of the food items listed (Table 4).

## Discussion

In this study the prevalence of overweight or obesity amongst these nursing students was higher than the national statistics reported from the SADHS 2003^3^ for the general South African population. However, 96.3% of the participants in the current study were Black persons and their overweight or obesity prevalence and trends very closely reflect national statistics, as well as trends, for the South African Black population >15yrs (27.2% for men; 56.2% for woman), reported by the SADHS 2003;^3^ as well as statistics (53.3% overweight or obese) reported for Black woman 25–34yrs in Mangaung, Free State.^29^ Only seven participants were underweight and, also in keeping with the findings of the SADHS^3^ and other South African studies,^30^ five of these were male.

In contrast, the percentages of overweight or obesity reported in this study were higher than those reported for Black participants at other South African universities in the past. For example, Morar *et al*.^31^ reported a prevalence of overweight or obesity of 24.3% for Black medical students at the University of Natal. Cilliers et al.^32^ reported a prevalence of overweight or obesity of 24.7% in Black first-year students at the University of Stellenbosch; whilst the prevalence of overweight or obesity amongst first-year female students was 25% at the University of Limpopo (Turfloop Campus) in 2000.^33^

Overweight or obesity figures of the Black nursing students in the current study are however similar to statistics reported for students at universities or colleges in North America^34, 35^ (where similar statistics have also been reported amongst graduate health care professionals^20^), but was higher than those reported for some other developing countries in the Middle East^36^ and Asia.^37^ This might indicate that the problem of overweight or obesity amongst Black students in South Africa is escalating, possibly due to rapid urbanisation and nutrition and lifestyle transitions to Western patterns.

In the current study, two-thirds of the nursing students (65.2%) had waist circumferences that put them at risk for insulin resistance and the metabolic syndrome according to the guidelines of the New Joint Classification for the Diagnosis of Metabolic Syndrome.^5^ Moreover, 38.5% of the participants had a waist circumference indicating substantial risk for NCD (≥102cm for males; ≥88cm for females).^4^ The high levels of truncal obesity in these participants was also confirmed by 37.3% of participants who had waist-hip-ratios indicative of increased health risk (≥0.9 for males; ≥0.8 for females). These figures are similar to the 42% reported by the SADHS 1998^38^ (using waist-hip-ratio cut-off points of ≥0.9 for males; ≥0.8 for females); and higher than what was reported by the SADHS 2003 (but which used higher waist-hip-ratio cut-off points of ≥1.0 for males; ≥0.85 for females) for Black men (5.1%) and women (31.7%).^3^

Quantification of the 24-hour recalls revealed quite low energy intakes, which in the light of the levels of overweight or obesity in this group, raised suspicions that the 'flat-slope syndrome’ – which refers to the phenomenon that participants with high actual consumptions often underestimate the amount of food and drink they recall^21^ – may have occurred; and/or may reflect low activity levels (which were not assessed in this study). Analysis of the 24-hour recalls of these predominantly Black nursing students, according to the food groups on the USDA-FGP, revealed low intakes of vegetables, fruit and dairy, which is characteristic of the nutritional transition reported by numerous studies amongst urbanised Black South African communities.^1, 12^ These signs of nutritional transition were also evident from the food frequency questionnaires obtained from this group: most participants consumed margarine, oil or fat and sugar on a daily basis, whilst few consumed vegetables, fruit, fruit juice and milk on a daily basis. Interestingly, most of the participants were from rural areas (42.2%) or townships (37.3%) and resided at the university hostels. The fact that they were living on campus may have contributed to fast-track the nutrition transition.

Vegetables and fruits are sources of vitamins, minerals, fibre and numerous phytochemicals which offer protection against NCD, including cardiovascular disease, type 2 diabetes and cancer, by various mechanisms which include acting as anti-oxidants that scavenge free radicals, enhancing the performance of the liver detoxification enzyme systems, and suppressing cancer cell initiation and/or proliferation.^39^ The finding that only 2.5% of these participants ate at least three vegetables daily, that almost half the participants (42.2%) did not eat at least two portions of fruit or fruit juice per day, and that the overall frequency of the intake of vegetables and fruits was very low, therefore poses a serious health risk. The nutritional knowledge questionnaire revealed that most of the participants also did not know the daily recommended number of servings that should be consumed from the vegetable and fruit groups. Nutritional knowledge is believed to play an important role in promoting healthier eating practices, and increased knowledge of dietary guidelines has been positively linked to more healthy eating practices amongst college students.^40^

Osteoporosis is another chronic disease risk posed by the fact that 92.6% of the participants consumed less than two servings of milk or dairy products per day, whilst only 15.8% indicated that they consume milk daily. Once again, the nutritional knowledge questionnaire revealed that most participants (60.2%) did not know how much milk or dairy products they ought to consume. The prevalence of osteoporosis is on the rise in SA – even amongst the Black population who is less prone to low bone density than Whites and Asians.^41^ The majority of the participants in the current study were in their twenties and had not reached peak bone density (at around 30 years) yet. Reaching an optimum peak bone density is considered one of the best ways to counteract the early onset of osteoporosis, and consuming adequate amounts of milk or dairy products is considered the most practical, effective and relatively inexpensive (when compared to the cost of supplements) way to acquire adequate calcium to optimise bone density.^42^ As the majority of the participants were Black South Africans, a high level of lactose intolerance may be expected amongst them.^43^ However, amongst South African Black persons, the use of fermented buttermilk, such as *amazi* which contains low levels of lactose, is part of the traditional eating habits.^43^ When students in a recent study (2010 – as yet unpublished results) amongst students on the University of the Free State (where 55% of students were Black students in 2010), were however asked to rank their preferences for foods that they would buy if available and affordable on campus, *amazi* and other traditional foods were ranked surprisingly low. This may confirm that the food preferences of Black South African students at University are westernised. A recent study confirmed a decrease in calcium intake with urbanisation amongst Black woman in the North West Province of South Africa.^41^ Weight-bearing exercise and other osteoprotective factors were not assessed in the current study.

Both the 24-hour recalls and the food frequency questionnaires pointed out high intakes of fat and sugar or sweets, although most participants were knowledgeable about the fact that these foods should be consumed sparingly. A practical way of cutting back on total and saturated fat intake is by substituting full cream milk with low fat or skim milk, however 76.5% of the participants reported that they do not use low fat or skim milk. Some significant associations could also be shown between higher fat and sugar intake in the overweight or obese participants compared to normal and/or underweight participants.

Self-reported alcohol intakes (Table 3) were within prudent guidelines.^27^ Most participants (73%) reported not using any alcohol and less than 1% reported daily consumption of alcohol. This is in contrast with reports of high alcohol consumption amongst university or college students in South Africa (2010 – yet unpublished study amongst students at the University of the Free State) and abroad^44^ yet underreporting is a possibility.

## Recommendations

Studies show that doctors and nurses are more likely to discuss weight, diet and lifestyle issues with their patients and use strategies to prevent obesity in patients if they themselves have a normal BMI.^20^ Literature also identifies lack of nutritional knowledge and confidence on the part of the health care provider as significant barriers which prevent health care workers from offering dietary support.^18, 19^ The fact that almost half of the nursing students in this study were overweight or obese and many lacked basic nutritional knowledge, may therefore impact negatively on their efficacy as future health care professionals. With rates of obesity and associated risks for NCD soaring in South Africa, universities or colleges are today faced with the significant challenge of how to meet these barriers and equip health care providers to support healthy choices and address risk factors in patients whilst primary prevention is still possible. The learning content of the training that nurses and other health professionals receive regarding nutrition and lifestyle should be critically reappraised towards this end.

However, knowledge of good eating habits and a healthy lifestyle is not necessarily enough to motivate health care professionals to practice what they (ought to) preach. Creating an environment that is supportive of healthy eating habits and an active lifestyle, as well as requiring health care students to show competence at applying these principles in their own lives, may go a long way towards achieving this aim. Future research should be directed at finding the most appropriate ways in which to create such structures for different student populations in different cultural settings. This would at least empower the future health care professionals to take charge of their own health, which may increase their efficacy in dealing with nutrition, health and weight issues in their patients.

## Conclusions

These South African nursing students, mainly from rural areas and townships, living in hostels at the University of Fort Hare, Eastern Cape, had a high prevalence of overweight and obesity, poor eating habits and inadequate knowledge on key nutritional issues, which puts them at risk for chronic diseases of lifestyle. As future health care professionals, this may impact negatively on their efficacy as health ambassadors to the public.
